# Introduction to the *Toxins* Special Issue “Ricin Toxins”

**DOI:** 10.3390/toxins12010013

**Published:** 2019-12-27

**Authors:** Nilgun E. Tumer

**Affiliations:** Department of Plant Biology, School of Environmental and Biological Sciences, Rutgers University, New Brunswick, NJ 08901-8520, USA; tumer@sebs.rutgers.edu; Tel.: +1-848-932-6359

Ricin toxin isolated from the castor bean (*Ricinus communis*) is one of the most potent and lethal molecules known. Castor beans are processed worldwide on an industrial scale for the castor oil. Ricin, a byproduct of castor oil, is a real threat for bioterrorism and for biological warfare, especially when dispersed by aerosol. There are no FDA approved vaccines or therapeutics to protect against ricin or the related Shiga toxins, which cause food poisoning and dysentery in millions of people around the world. Ricin is a type II ribosome inactivating protein (RIP), which consists of an active A chain (RTA) covalently linked to a cell binding B chain (RTB). RTA inhibits protein synthesis by removing a specific adenine from the highly conserved α-sarcin/ricin loop (SRL) in the large rRNA and inhibits protein synthesis. RTA-antibody complexes have been explored as immunotoxins against cancer cells. A thorough understanding of how ricin enters cells and traffics to the ribosome, how it inactivates ribosomes with near perfect efficiency, how it induces inflammatory signaling pathways, and programmed cell death is critical for understanding the complexity of ricin and for reducing its toxicity. The eight articles published in this issue address these research needs and provide important insights into the mechanisms of the toxicity of ricin. They will contribute to the design of therapies against intoxication by ricin and related toxins.

Polito et al. [1] review the history of ricin starting from its use in traditional and folk medicine and highlight the research milestones in the characterization of enzymatic activity, structure, toxicity, and medical applications [1]. Ricin is rapidly internalized and catalytic amounts are needed to inhibit protein synthesis. It has been used as a powerful tool to understand intracellular trafficking and cell death pathways. Sowa-Rogozinska et al. [2] review the current knowledge about the intracellular transport of ricin and identification of host factors that facilitate transport to increase our understanding of the mechanism of the cytotoxicity of ricin. This review summarizes medical applications of ricin and highlights its role as a valuable component of immunotoxins against cancer [2].

Previous studies identified the host target of ricin as the ribosomal P stalk [3,4] and showed that binding to the P stalk is necessary for depurination of the SRL by RTA on intact ribosomes [5]. The eukaryotic P stalk contains P0 protein and two P1–P2 dimers with identical C-terminal sequences, which are critical for interaction with the translation factors and factor dependent GTP hydrolysis. Ricin binds to the C-termini of the human P1–P2 dimer, which represents the smallest component of the eukaryotic stalk [6]. Grela et al. [7] present the current understanding of the structure and function of the ribosomal stalk and the consequence of ricin dependent depurination of the SRL on ribosome performance and translation.

Small molecules that can enter and rescue intoxicated cells by inactivating intracellular ricin are highly sought after as countermeasures. Although small-molecule RIP inhibitors have been identified, none of them exhibited potent protection against RIPs. Li et al. addressed if peptides mimicking the conserved C-terminal sequences of P proteins will inhibit the activity of RTA by preventing its interaction with the ribosome [8]. They show that these peptides interact with the ribosome binding site of RTA and inhibit the activity of RTA by disrupting its interaction with ribosomes [8]. These results establish the ribosome binding site of RTA as a new target for inhibitor discovery [8].

Ricin inhalation causes acute lung injury characterized by a massive inflammatory response. Hodges et al. [9] evaluated the cell death modulatory activity of cytokines in ricin toxicity in human lung epithelial cells and showed that tumor necrosis factor (TNF) family cytokines induce distinct cell death pathways when administered in combination with ricin [9]. Targeting these cell death pathways may lead to novel therapeutic approaches to ricin toxicity [9]. The use of neutralizing antibodies is a promising post-exposure treatment against ricin intoxication. Falach et al. [10] generated equine derived antibodies against ricin for post exposure treatment. They generated an inactivated toxin and constructed monomerized ricin antigen by irreversible reduction of the A and B subunits. Immunization of a horse with the monomerized toxin yielded high titers of neutralizing antibodies. Passive immunization of mice with equine derived F(ab’)_2_ based antitoxin conferred protection against a lethal intranasal ricin challenge [10].

Ricin is a therapeutic agent and a potential threat to public health and safety. Several methods to detect ricin have been developed; however, each method has its limitations [11]. Innovative assays for toxin detection and mitigation are needed. Micro RNA (miRNA) profiles can help understand ricin toxicity mechanisms and could serve as potential biomarkers for ricin intoxication. Pillar et al. [12] investigate the effect of pulmonary exposure of mice to ricin on miRNA expression profiles in mouse lungs. They show significant changes in the lung tissue expression levels of miRNAs involved in innate immunity pathways. They confirm these findings by gene expression analysis and show activation of immune regulation pathways and immune cell recruitment after ricin exposure [12]. Sousa et al. [13] describe an accelerated solvent extraction (ASE) method followed by matrix-assisted laser desorption/ionization time-of-flight mass spectrometry (MALDI-TOF-MS) and MALDI-TOF-MS/MS for extraction and detection of ricin in forensic samples. This method could also detect ricin in gamma-irradiated samples [13].

The papers in this issue provide readers with a better understanding of ricin trafficking, ribosome binding, SRL depurination, cell signaling, toxicity, and ricin detection mechanisms and identify new targets that may be useful in the development of ricin antidotes.
